# Supplementary material for: The NCBI Comparative Genome Viewer (CGV) is an interactive visualization tool for the analysis of whole-genome eukaryotic alignments
Source: PLoS Biol. 2024 May 7;22(5):e3002405. doi: 10.1371/journal.pbio.3002405 (PMC11101090; doi:10.1371/journal.pbio.3002405)
Supplement: S1 Appendix — CGV’s ideogram view displays best-placed alignments by default. Non-best placed alignments can be added from the “Adjust Your View” configure options (Fig 1F). (A) CGV view of whole-genome alignment of Arabidopsis thaliana and A. suecica, a polyploid hybrid of A. thaliana and A. arenosa [37]. The default view shows regions of similarity between A. thaliana and the A. thaliana subgenome of A. suecica. Including non-best placed alignments reveals additional alignment segments (marked by red boxes) that correspond to alignment between A. thaliana and the A. arenosa subgenome of A. suecica. https://www.ncbi.nlm.nih.gov/genome/cgv/browse/GCA_019202805.1/GCF_000001735.4/48965. (B–E) CGV alignments between human and chimpanzee (B, C, and E) and human and bonobo (D) genome assemblies in regions that contain local segmental duplications. When non-best placed alignments are shown, additional alignment segments are displayed that identify potential additional copies of BMPR2 (B), EIF4A3 (C and D), or EYS related gene sequence (E). EIF4A3 was previously reported to have multiple copies in both chimpanzee and bonobo relative to the human genome [38]. (B). https://www.ncbi.nlm.nih.gov/genome/cgv/browse/GCF_028858775.1/GCF_000001405.40/35595/0#NC_072400.1:104175446-105102154/NC_000002.12:202185329-203061800/size=1000,firstpass=0. (C). https://www.ncbi.nlm.nih.gov/genome/cgv/browse/GCF_028858775.1/GCF_000001405.40/35595/0#NC_072415.1:91293196-91791317/NC_000017.11:80086216-80514871/size=1000,firstpass=0. (D). https://www.ncbi.nlm.nih.gov/genome/cgv/browse/GCF_029289425.1/GCF_000001405.40/36375/9606#NC_073266.1:100957682-101607434/NC_000017.11:80031356-80681076/size=1000,firstpass=0. (E). https://www.ncbi.nlm.nih.gov/genome/cgv/browse/GCF_028858775.1/GCF_000001405.40/35595/0#NC_072404.1:68562328-78605127/NC_000006.12:62860419-76648142/size=1000,firstpass=0. (PDF) [file pbio.3002405.s001.pdf]

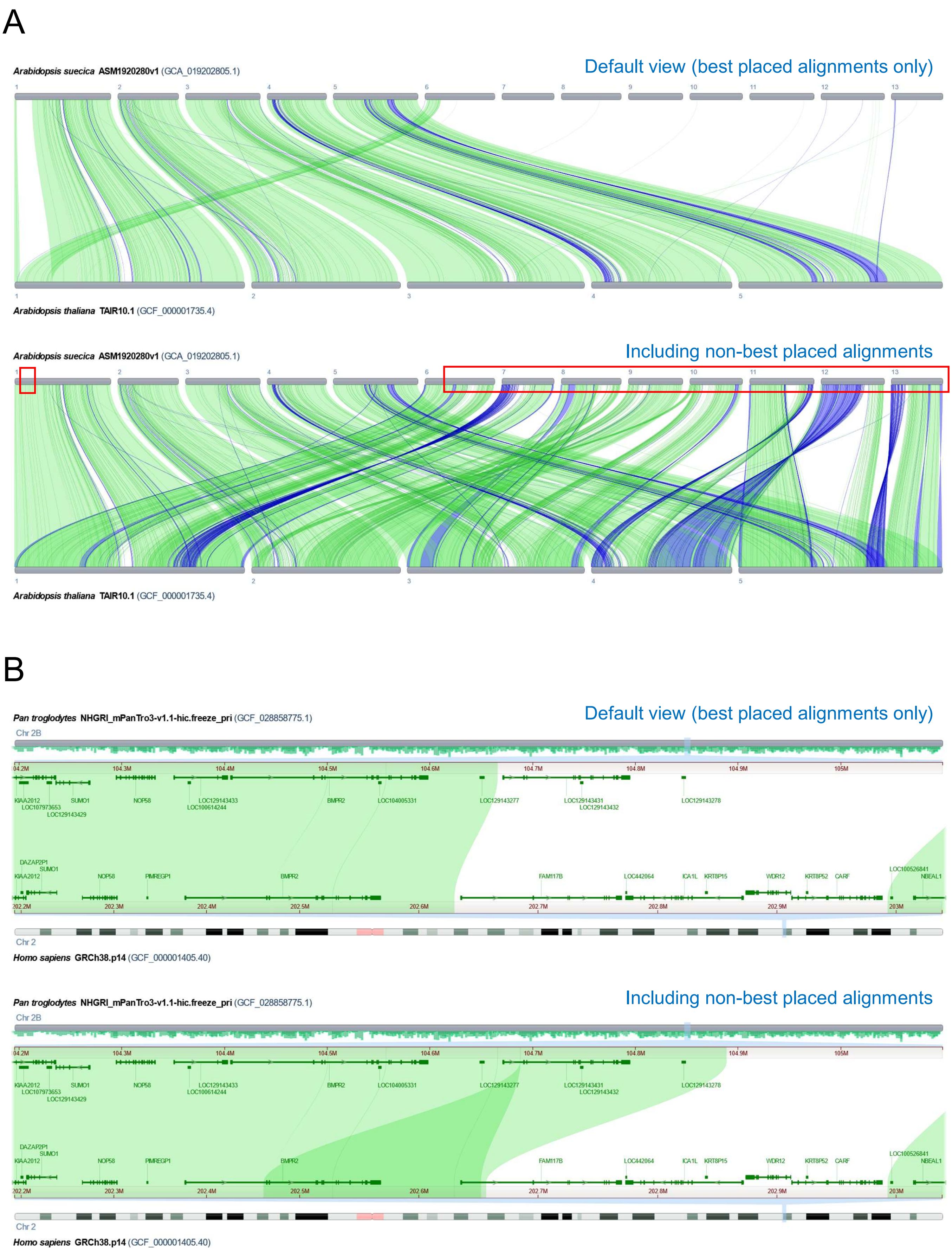
A
Arabidopsis suecica ASM1920280v1 (GCA_019202805.1)
Default view (best placed alignments only)
Arabidopsis thaliana TAIR10.1 (GCF_000001735.4)
Arabidopsis suecica ASM1920280v1 (GCA_019202805.1)
Including non-best placed alignments
Arabidopsis thaliana TAIR10.1 (GCF_000001735.4)
B
Pan troglodytes NHGRI_mPanTro3-v1.1-hic.freeze_pri (GCF_028858775.1)
Default view (best placed alignments only)
Chr 2B
104.3M
104.4M
104.5M
104.6M
104.7M
104.8M
104.9M
105M
KIAA2012
LOC107973653
LOC129143429
SUMO1
NOP58
LOC129143433
LOC100614244
BMPR2
LOC104005331
LOC129143277
LOC129143431
LOC129143432
LOC129143278
DAZAP2P1
SUMO1
KIAA2012
NOP58
PIMREGP1
BMPR2
FAM117B
LOC442064
ICA1L
KRT8P15
WDR12
KRT8P52
CARF
LOC100526841
NBEAL1
202.2M
202.3M
202.4M
202.5M
202.6M
202.7M
202.8M
202.9M
203M
Chr 2
Homo sapiens GRCh38.p14 (GCF_000001405.40)
Pan troglodytes NHGRI_mPanTro3-v1.1-hic.freeze_pri (GCF_028858775.1)
Including non-best placed alignments
Chr 2B
Chr 2
Homo sapiens GRCh38.p14 (GCF_000001405.40)

C

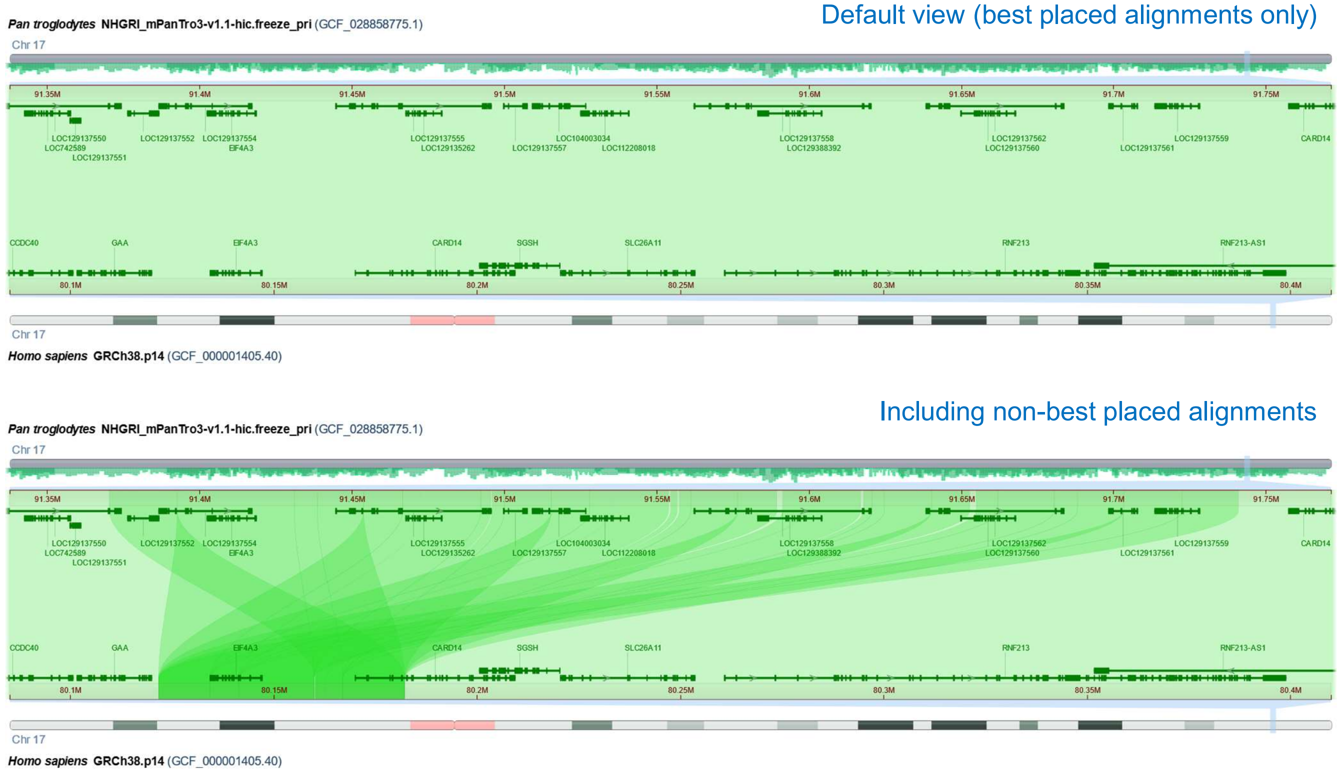
Default view (best placed alignments only)
Pan troglodytes NHGRI_mPanTro3-v1.1-hic.freeze_pri (GCF_028858775.1)
Chr 17
LOC129137550
LOC742589
LOC129137551
LOC129137552
LOC129137554
EIF4A3
LOC129137555
LOC129135262
LOC129137557
LOC104003034
LOC112208018
LOC129137558
LOC129388392
LOC129137562
LOC129137560
LOC129137561
LOC129137559
CARD14
CCDC40
GAA
EIF4A3
CARD14
SGSH
SLC26A11
RNF213
RNF213-AS1
Chr 17
Homo sapiens GRCh38.p14 (GCF_000001405.40)
Including non-best placed alignments
Pan troglodytes NHGRI_mPanTro3-v1.1-hic.freeze_pri (GCF_028858775.1)
Chr 17
Chr 17
Homo sapiens GRCh38.p14 (GCF_000001405.40)

D

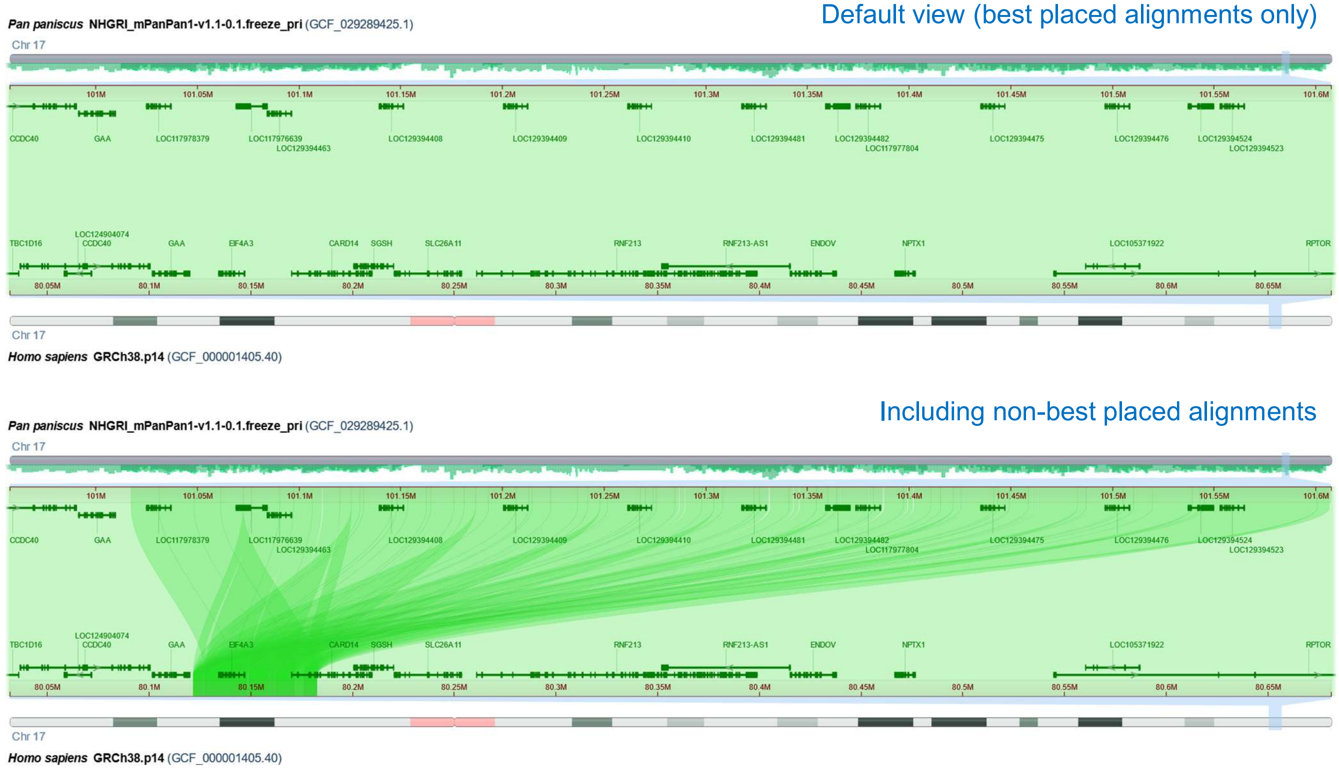
Default view (best placed alignments only)
Pan paniscus NHGRI_mPanPan1-v1.1-0.1.freeze_pri (GCF_029289425.1)
Chr 17
CCDC40
GAA
LOC117978379
LOC117976639
LOC129394463
LOC129394408
LOC129394409
LOC129394410
LOC129394481
LOC129394482
LOC117977804
LOC129394475
LOC129394476
LOC129394524
LOC129394523
TBC1D16
LOC124904074
CCDC40
GAA
EIF4A3
CARD14
SGSH
SLC26A11
RNF213
RNF213-AS1
ENDOV
NPTX1
LOC105371922
RPTOR
Chr 17
Homo sapiens GRCh38.p14 (GCF_000001405.40)
Including non-best placed alignments
Pan paniscus NHGRI_mPanPan1-v1.1-0.1.freeze_pri (GCF_029289425.1)
Chr 17
Chr 17
Homo sapiens GRCh38.p14 (GCF_000001405.40)

E

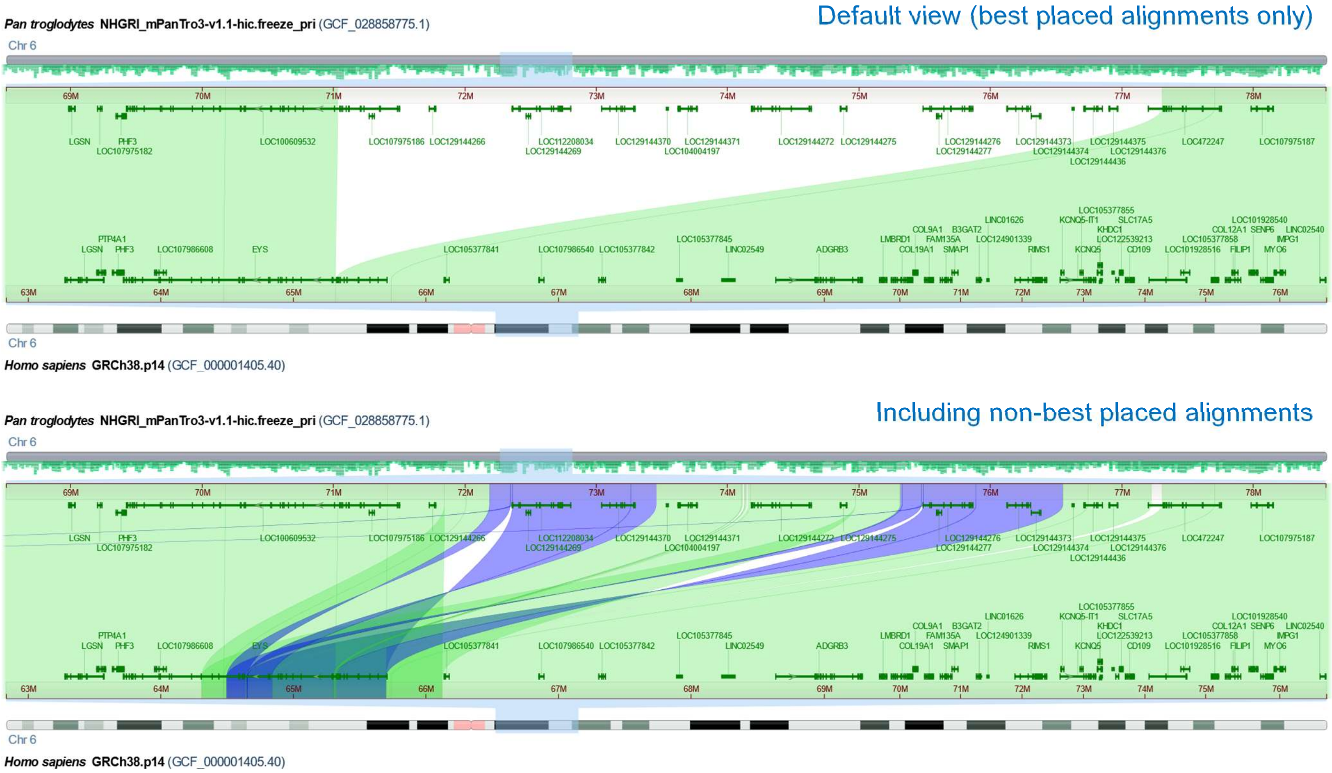

Pan troglodytes NHGRI_mPanTro3-v1.1-hic.freeze_pri (GCF_028858775.1)
Default view (best placed alignments only)
Chr 6
Chr 6
Homo sapiens GRCh38.p14 (GCF_000001405.40)
Pan troglodytes NHGRI_mPanTro3-v1.1-hic.freeze_pri (GCF_028858775.1)
Including non-best placed alignments
Chr 6
Chr 6
Homo sapiens GRCh38.p14 (GCF_000001405.40)
