# Supplementary material for: The NCBI Comparative Genome Viewer (CGV) is an interactive visualization tool for the analysis of whole-genome eukaryotic alignments
Source: PLoS Biol. 2024 May 7;22(5):e3002405. doi: 10.1371/journal.pbio.3002405 (PMC11101090; doi:10.1371/journal.pbio.3002405)
Supplement: Attachment — Submitted filename: CGV-PLoS Biology- Response to Reviewers.docx [file pbio.3002405.s004.docx]

**Response to Reviewers**

*Reviewer #1:*

*In this manuscript Rangwala et al. present a new tool for visualising whole genome comparisons using precomputed alignments of publicly available assemblies for hundreds of species across the tree of life.*

*They outline some widely used tools and visualisation methods for displaying genomic data and outline limitations with respect of assembly-assembly alignments. They show how the Comparative Genome Viewer presents alignment data in a clear and accessible manner on a platform (browser) open to all users, they do a step by step guide to using CGV and also highlight some interesting use cases.*

*They talk at length about front and back end set up and design choices and implementation for a good user experience, they also cover some usage limitations and potential new features to implement.*

*This is a nice well written paper, figures are clear and concise and relevant to the manuscript. There are no big concerns about the presented data and there is definitely space for a tool such as this.*

*Not something to address in the paper but two thirds of genomes in the alignment set are animals with 20% of the total being mammalian, this is not representative of the publicly available assemblies in GenBank. In addition, it would be worth having some assembly quality metric to help the end user to better understand the genome data, as the authors raise repeatedly the potential for there to be assembly errors.*

**Thank you for your comments. We’ve been adding alignments to CGV largely in response to user requests, which is why they likely aren’t proportionately representative of what is available in GenBank.**

**Regarding reporting assembly quality, users can navigate from CGV to NCBI Datasets assembly record pages, where they can find measures of assembly quality such as BUSCO scores and scaffold and contig N50s; e.g.** [**https://www.ncbi.nlm.nih.gov/datasets/genome/GCF_009873245.2/**](https://www.ncbi.nlm.nih.gov/datasets/genome/GCF_009873245.2/)**.**

*I do recommend the paper for publication.*

Reviewer #2:

*Rangwala and colleagues have built a useful and user-friendly interactive visualization tool for pairwise comparison of genomic synteny and alignments. Having worked to build one of these myself, I commend the authors on the simplicity of the user-facing operations and the variety of downstream analytical tools. I have a few thoughts that I hope will improve the utility of the tool, or alternatively better contextualize the scale/scope of the results presented in the manuscript.*

*It looks like the synteny finding algorithm doesn't deal well with whole-genome duplications. As far as I could tell, there is only one comparison available in the CGV that crosses a whole-genome duplication, that of Xenopus laevis vs. X. tropicalis (*[*https://www.ncbi.nlm.nih.gov/genome/cgv/browse/GCF_000004195.4/GCF_017654675.1/38475/8355*](https://www.ncbi.nlm.nih.gov/genome/cgv/browse/GCF_000004195.4/GCF_017654675.1/38475/8355)*), and the alignments therein do not look good. Since the WGD is on the X. laevis branch (Fig. 2 here:* [*https://gcc02.safelinks.protection.outlook.com/?url=https%3A%2F%2Fwww.nature.com%2Farticles%2Fnature19840&data=05%7C02%7Csanjida.rangwala%40nih.gov%7Cd1125e14d982447e5bbf08dc15b3a7dd%7C14b77578977342d58507251ca2dc2b06%7C0%7C0%7C638409108561712472%7CUnknown%7CTWFpbGZsb3d8eyJWIjoiMC4wLjAwMDAiLCJQIjoiV2luMzIiLCJBTiI6Ik1haWwiLCJXVCI6Mn0%3D%7C3000%7C%7C%7C&sdata=9cgLLpSnZj3nOhvPy2%2FTgi7wyWyL6jvsww7gp394Puo%3D&reserved=0)*](https://gcc02.safelinks.protection.outlook.com/?url=https%3A%2F%2Fwww.nature.com%2Farticles%2Fnature19840&data=05%7C02%7Csanjida.rangwala%40nih.gov%7Cd1125e14d982447e5bbf08dc15b3a7dd%7C14b77578977342d58507251ca2dc2b06%7C0%7C0%7C638409108561712472%7CUnknown%7CTWFpbGZsb3d8eyJWIjoiMC4wLjAwMDAiLCJQIjoiV2luMzIiLCJBTiI6Ik1haWwiLCJXVCI6Mn0%3D%7C3000%7C%7C%7C&sdata=9cgLLpSnZj3nOhvPy2%2FTgi7wyWyL6jvsww7gp394Puo%3D&reserved=0))*, both homeologs should be similarly related to X. tropicalis, so primary and secondary alignments have no meaning with regard to the homeologs. Yet you can only see the duplication with the inclusion of secondary alignments. I understand that finding synteny from whole-genome alignments that cross a WGD is difficult (unlike with CDS, which is simple), but it is doable with the right context or algorithm. I'd strongly suggest either re-writing the algorithm to handle these cases, or state up front*
*that the CGV cannot handle comparisons of variable ploidy. If you disagree with me and think that the GCV can handle WGDs, please include examples of simple comparisons, like Xenopus laevis --> human, wheat --> Brachypodium distachyon and Brassica napus (oilseed rape) --> Arabidopsis thaliana, and show that genome coverage is equally good for all homeologs.*

*Depending on the response to my above comment regarding WGDs, I think the statement that CGV is something that is useful for eukaryotes in general is not accurate. Really, it appears that it is only useful for single-copy genomes. I bring this point up because all of the plant species included in the CGV only extend to other genomes that coalesce before a whole-genome duplication event. However, in many cases, a user would want to use this tool in plants like they would in animals - to explore regions of interest between a genome and its related model system. Nearly all alignments from crops to the major plant models (Arabidopsis and Brachypodium) cross at least one WGD. There are ~5x more plant species than vertebrates … the CGV would not be useful for the vast majority of these.*

**Thank you for your thoughtful analysis. Please note that, in the example of *Xenopus laevis*, the Fig 2 that was referenced above also shows that the two subgenomes L and S are diverged and the L subgenome is slightly closer to *X. tropicalis* than the S subgenome (Fig 2b). Accordingly, our alignment algorithm shows a bias towards L subgenomes in the selection of best-placed alignments (default view). Users can view alignments to both subgenomes by choosing the option to “include non-best place alignments” in the CGV ideogram view. The CGV dot-plot view also shows all alignments (**[**https://www.ncbi.nlm.nih.gov/genome/cgv/plot/GCF_000004195.4/GCF_017654675.1/38475/8355**](https://www.ncbi.nlm.nih.gov/genome/cgv/plot/GCF_000004195.4/GCF_017654675.1/38475/8355)**). Users can browse details of all alignments in a genomic region by following the GDV links within each alignment in CGV.**

**Our alignment logic aims to identify alignments representing reciprocally the best homologous or orthologous genomic regions while the rest are kept as non-best placed alignments. This works best for relatively older whole genome duplications (e.g., mesopolyploids) or for allopolyploids where subgenomes can be distinguished. In these cases, primary alignments should correspond to the orthologous regions, while additional homeologous regions will be shown in secondary alignments when users activate the option to “include non-best placed alignments.” Please refer to example plant diploid vs. allopolyploid alignments, e.g. *Coffea arabica* vs *C. canephora* (**[**https://ncbi.nlm.nih.gov/genome/cgv/browse/GCA_900059795.1/GCF_003713225.1/37165/13443**](https://ncbi.nlm.nih.gov/genome/cgv/browse/GCA_900059795.1/GCF_003713225.1/37165/13443)**) or *Arabidopsis thaliana* vs *A. suecica* (**[**https://ncbi.nlm.nih.gov/genome/cgv/browse/GCA_019202805.1/GCF_000001735.4/48965/3702**](https://ncbi.nlm.nih.gov/genome/cgv/browse/GCA_019202805.1/GCF_000001735.4/48965/3702)**). The latter comparison can be viewer in the new S1 Appendix supplemental file provided in the updated manuscript submission.**

**We also added *A. thaliana* vs *Brassica napus*, where the *A. thaliana* genome showed preference to the A subgenome (derived from *B. rapa*) in the best-placed alignments (**[**https://www.ncbi.nlm.nih.gov/genome/cgv/browse/GCF_000001735.4/GCF_020379485.1/49185/3702**](https://www.ncbi.nlm.nih.gov/genome/cgv/browse/GCF_000001735.4/GCF_020379485.1/49185/3702)**), while the remaining are captured as the non-best placed alignments, which can be further explored in GDV using links available for each alignment in CGV.**

**Our alignment logic, designed to work without the need for gene model annotations, is not ideal for recent whole genome duplications with undifferentiated subgenomes (e.g., *A. thaliana* vs. *Camelina sativa*) or genome pairs with large ploidy level and size differences (e.g., sorghum vs. sugar cane and *Brachypodium* vs. wheat). In these cases, different alignment logic that prioritizes protein-coding gene order synteny would be more appropriate. We recognize this as an area for future improvement. As a result, we avoid providing alignments for genomes with recent whole genome duplications since these may be unreliable. We have added clarification to the beginning of the Results and the Discussion section of the manuscript (lines 124-128; 376-382).**

*The CGV is not going to be useful for comparisons of very diverged species that lack solid synteny. In amniotes, synteny is remarkably conserved and there still is ~50% genome coverage between Human and Xenopus:* [*https://www.ncbi.nlm.nih.gov/genome/cgv/browse/GCF_000004195.4/GCF_000001405.40/46255/9606#NC_030677.2//size=10000*](https://www.ncbi.nlm.nih.gov/genome/cgv/browse/GCF_000004195.4/GCF_000001405.40/46255/9606#NC_030677.2//size=10000) *cover ~50% of the genome). However, as you go deeper (e.g. humans to zebrafish) or with less conserved genomes (*[*https://www.ncbi.nlm.nih.gov/genome/cgv/browse/GCF_000002335.3/GCF_023101765.2/42855/7070*](https://www.ncbi.nlm.nih.gov/genome/cgv/browse/GCF_000002335.3/GCF_023101765.2/42855/7070)*), the utility breaks down. While this limitation is obvious to someone well-versed in comparative genomics, it won't be to the vast majority of users and needs to be stated up front.*

**Thank you. This limitation is acknowledged in the Discussion section of the manuscript (lines 376-382). We have now also stated it up front near the beginning of the Results section (lines 121-123).**

*It is stated that ~700 genomes are available - this makes it seem like one could compare all of these genomes to each other. This is not the case. The majority of the pre-computed alignments are to a very restricted set of genomes, mostly within the same species. I think this is fine, but this limitation needs to be stated clearly up front. Perhaps instead of having all genomes together in one dropdown, have a "project" or whatever selection that gets you to all genomes that can be compared?*

**Thank you for your suggestion. We have added some additional clarification that the alignments we provide are mainly within the same species or closely related species (lines 135-138).**

*Genomes have variable naming conventions, and this can produce comparisons that look messy, but are actually very syntenic. The rat/mouse comparison illustrates this: there are some rearrangements, but these are hard to see since syntenic chromosomes are not stacked. The Phytozome comparative genomics viewer (*[*https://gcc02.safelinks.protection.outlook.com/?url=https%3A%2F%2Fphytozome-next.jgi.doe.gov%2Ftools%2Fsynteny&data=05%7C02%7Csanjida.rangwala%40nih.gov%7Cd1125e14d982447e5bbf08dc15b3a7dd%7C14b77578977342d58507251ca2dc2b06%7C0%7C0%7C638409108561716428%7CUnknown%7CTWFpbGZsb3d8eyJWIjoiMC4wLjAwMDAiLCJQIjoiV2luMzIiLCJBTiI6Ik1haWwiLCJXVCI6Mn0%3D%7C3000%7C%7C%7C&sdata=mY4a0LuwLfgxuV2nZ%2FOsSdNCUoT5irSc6MOqCeKIL6o%3D&reserved=0*](https://gcc02.safelinks.protection.outlook.com/?url=https%3A%2F%2Fphytozome-next.jgi.doe.gov%2Ftools%2Fsynteny&data=05%7C02%7Csanjida.rangwala%40nih.gov%7Cd1125e14d982447e5bbf08dc15b3a7dd%7C14b77578977342d58507251ca2dc2b06%7C0%7C0%7C638409108561716428%7CUnknown%7CTWFpbGZsb3d8eyJWIjoiMC4wLjAwMDAiLCJQIjoiV2luMzIiLCJBTiI6Ik1haWwiLCJXVCI6Mn0%3D%7C3000%7C%7C%7C&sdata=mY4a0LuwLfgxuV2nZ%2FOsSdNCUoT5irSc6MOqCeKIL6o%3D&reserved=0)*) handles this with the "sort scaffold" toggle. Incorporating this here would be helpful.*

**Thank you for your suggestion. We will take it into consideration as we continue to improve this tool in response to feedback from our users. Up until now, we have opted to order chromosomes in the numerical order provided by the original submitter of the genome assembly. By faithfully representing the submitted assembly, we can highlight to the user where the assembly chromosome numbering (or orientation) could be improved and made more consistent among related genomes.**

*Reviewer #3:*

*In this manuscript, the authors introduce the Comparative Genome Viewer (CGV), a web-based pairwise whole-genome alignment visualization tool hosted by the NCBI. The tool allows visualization of ~700 pre-computed pairwise alignments from eukaryotics species. The pairwise alignments are computed using BLAST, LASTZ, or imported from other sources and then post-processed using a custom algorithm. The visualization uses an existing "stacked linear browser" approach and includes gene annotations as tracks for both genome assemblies. The tool has links out to views within the existing Genome Data Viewer (GDV) for browsing within single genomes.*

*Overall, the CGV is an intuitive tool that provides access to a large number pairwise whole genome alignments. It should be useful to researchers needing to examine orthologous regions from previously published genome assemblies. It is not designed to handle newly-sequenced unpublished genomes, and thus generators of such data will need to use other tools for initial comparative analysis of their genomes. I have just one major critique of the manuscript with respect to its comparisons to existing tools and handful of minor comments.*

*Major comments:*

*1. There are several other large-scale providers of pairwise genome alignments and associated visualization tools. For example, the UCSC Genome Browser has a chain/net track that allows for visualizing pairwise alignments with another genome and can easily show rearranged segments (contradicting lines 67-70). The Ensembl website also hosts many pairwise alignments and has dedicated visualizations for viewing them. This manuscript should more strongly make the case for why an additional tool is needed in this space. For example, a figure showing how the these different tools display the alignment of the same region could help to show the strengths and weaknesses of these tools.*

**We are aware of UCSC Genome Browser’s “multiZ” style and chain/net tracks. We have also explored the different views available in Ensembl. Our GDV browser also displays assembly-assembly data as linear alignment tracks, e.g.** <https://www.ncbi.nlm.nih.gov/genome/gdv/browser/genome/?cfg=NCID_1_44919507_130.14.18.128_9146_1709585090_4030585770> **(URL expires in 90 days).**

**The advantage of CGV’s two-dimensional view is that it readily communicates how chromosomes in one assembly are evolutionarily related to chromosome regions in a different assembly, thus allowing the researcher to more easily understand relative genome translocations and breaks in syntenic blocks. This information is more difficult to parse from linear genome browser-style tracks. We have clarified these distinctions in the Introduction of the manuscript, while also adding additional information about other tools used in comparative genomics, including UCSC’s chain/net tracks and Ensembl’s two-dimensional synteny view (lines 60-73).**

*Minor comments:*

*2. Going to dotplot view from ideogram view puts you back at whole-genome level not the regions currently being viewed in ideogram view, which is counterintuitive.*

**Thank you for your close investigation of the CGV application! This application is still in active development in response to user feedback and usability testing.**

**Because the ideogram view allows for more granular zooming than the dotplot view, it is not currently possible to zoom to the exact region in the dotplot as the ideogram view. When and if we add the ability to zoom further within the dotplot, it will become possible to navigate from the dotplot to a specific region in the ideogram, and vice versa, and we will be able to update the behavior of the application.**

*3. The download button downloads entire whole-genome alignment, not the currently displayed part of the alignment.*

**Thank you for this feedback. We will look at this aspect of the user interface more closely as we make further refinements and undergo more application testing with a broad spectrum of users.**

*4. It is unclear how to download in XLSX format - this option was always greyed out even when zooming in to small regions. It is also unclear what information this format conveys.*

**Thank you for this feedback. The XLSX and ‘text report’ files are only available for same-species or closely related assembly alignments produced by our BLAST-based alignment workflow. They are not available for LASTZ-based alignments. Refer to** [**https://ncbi.nlm.nih.gov/genome/cgv/browse/GCA_947599735.1/GCF_000001635.27/46365/1009**](https://ncbi.nlm.nih.gov/genome/cgv/browse/GCA_947599735.1/GCF_000001635.27/46365/1009) **for an example alignment that is able to export XLSX and text files.**

*5. Can other data tracks other than gene annotations be added?*

**CGV only displays gene annotation, either from RefSeq or from the submitter if RefSeq annotation is not available for an assembly. The user can navigate to our GDV genome browser to add additional tracks, including user-uploaded tracks or track hubs.**

*6. I found the base level view interface difficult to understand, particulary in how it interacts with the main interface. Some further desciription or documentation of this interface would be helpful.*

**Thank you for this feedback. This sequence level view is a preliminary implementation, and we plan to improve this interface in the future. Because it may change, we did not provide details on this view panel in this current manuscript. There is some documentation about this view in our Help documentation (**[**https://ncbi.nlm.nih.gov/genome/cgv/cm/cgv/more_info#Sequence_differences**](https://ncbi.nlm.nih.gov/genome/cgv/cm/cgv/more_info#Sequence_differences)**).**

*7. Figures in this manuscript need to be at higher resolution as many details are blurry - given that CGV can export in SVG this should be easily achievable.*

**Thank you for this feedback. We have updated the figure files in our manuscript re-submission to improve the resolution.**

*8. A custom approach is described for processing initial alignments, however, no software is provided as an implementation. Ideally, this software should be made publicly available so that other researchers can evaluate the approach on other data.*

**We have provided a detailed description of how we generate and process assembly-assembly alignments for CGV (Materials and Methods; S1 Figure). This process is not entirely novel; the core alignments are generated using megaBLAST or LASTZ using previously published methods (Steinberg et al. 2014; Kirilenko et al. 2023) (refer to manuscript for citation details). The most notable modification, the use of WindowMasker to adjust the masking level, is available as part of the publicly available NCBI C++ toolkit (**[**https://www.ncbi.nlm.nih.gov/toolkit**](https://www.ncbi.nlm.nih.gov/toolkit)**).**

**Alignment post-processing was designed to make the alignments compatible with internal NCBI data processes, including our internal alignment storage system and our previously published Genome Data Viewer (GDV) browser. The processing logic was implemented as modifications to a legacy pipeline which has extensive database and internal library dependencies that preclude its compilation or use outside of NCBI infrastructure. Rebuilding the code in a way that is reusable outside of NCBI would require a major redesign that is outside the scope of this project. Because the core focus of the paper is on the CGV application itself, we believe that description provided in the Materials and Methods is appropriate for the readers of this manuscript.**

*9. Please cite and explain Mash distance at its first mention in the manuscript.*

**We have added the citation to the first mention of Mash in the manuscript (line 261-262). The text briefly describes the methodology.**

*10. Line 60 - perhaps mention IGV as it is a popular desktop-based genome browser*

**We have added a reference to IGV to the Introduction of the manuscript as suggested (line 58).**

*11. An example figure showing a non-best alignment with one region in one genome alignment to multiple regions in another would be helpful to understand how this visualization works.*

**Thank you for the suggestion. In a new supplemental file (S1 Appendix), we have provided examples of CGV views where showing non-best placed alignments reveals that a single copy gene in one assembly aligns to multiple regions in a second assembly. These examples represent genomic regions where a gene copy number expansion may have occurred in one assembly relative to another.**

*12. amylase family expansion example: it is unclear why there is an unaligned sequence segment in CHM13 if it contains additional copies of the alpha-amylase gene. Are these part of "next-best" alignments that are not shown? If not, why are they not part of an alignment?*

**The GRCh38 vs T2T-CHM13v2.0 alignments currently available in CGV were generated by the T2T/HPRC consortium using a minimap2-based process described at** [[**https://s3-us-west-2.amazonaws.com/human-pangenomics/T2T/CHM13/assemblies/chain/v1_nflo/v1_nflo_description.html**](https://s3-us-west-2.amazonaws.com/human-pangenomics/T2T/CHM13/assemblies/chain/v1_nflo/v1_nflo_description.html)](https://s3-us-west-2.amazonaws.com/human-pangenomics/T2T/CHM13/assemblies/chain/v1_nflo/v1_nflo_description.html)**. This process used rustybam to trim the alignments down to only reciprocal best placed alignments, dropping the non-best placed alignments in regions such as the alpha-amylase expansion. We have opted to use the T2T/HPRC alignments for consistency with other resources such as UCSC and Ensembl. This is currently the only alignment set shown in CGV that used this minimap2-based process. You can find the source of the alignment by clicking on the (i) symbol after the alignment description on the view page.**

**We have provided additional alignments examples in the new S1 Appendix in response to item 11 above. These alignments were generated using our in-house alignment process. In these examples, clicking on “include non-best placed alignments” shows alignments to additional gene homologs.**
